# Modulation of Functional Connectivity and Low-Frequency Fluctuations After Brain-Computer Interface-Guided Robot Hand Training in Chronic Stroke: A 6-Month Follow-Up Study

**DOI:** 10.3389/fnhum.2020.611064

**Published:** 2021-01-20

**Authors:** Cathy C. Y. Lau, Kai Yuan, Patrick C. M. Wong, Winnie C. W. Chu, Thomas W. Leung, Wan-wa Wong, Raymond K. Y. Tong

**Affiliations:** ^1^Department of Biomedical Engineering, The Chinese University of Hong Kong, Hong Kong, China; ^2^Brain and Mind Institute, The Chinese University of Hong Kong, Hong Kong, China; ^3^Department of Imaging and Interventional Radiology, Prince of Wales Hospital, The Chinese University of Hong Kong, Hong Kong, China; ^4^Department of Medicine and Therapeutics, Prince of Wales Hospital, The Chinese University of Hong Kong, Hong Kong, China; ^5^Department of Psychiatry and Biobehavioural Sciences, University of California, Los Angeles, Los Angeles, CA, United States

**Keywords:** stroke, rehabilatation robotics, functional magnet resonance imaging, brain-computer interface, fractional amplitude low-frequency fluctuations

## Abstract

Hand function improvement in stroke survivors in the chronic stage usually plateaus by 6 months. Brain-computer interface (BCI)-guided robot-assisted training has been shown to be effective for facilitating upper-limb motor function recovery in chronic stroke. However, the underlying neuroplasticity change is not well understood. This study aimed to investigate the whole-brain neuroplasticity changes after 20-session BCI-guided robot hand training, and whether the changes could be maintained at the 6-month follow-up. Therefore, the clinical improvement and the neurological changes before, immediately after, and 6 months after training were explored in 14 chronic stroke subjects. The upper-limb motor function was assessed by Action Research Arm Test (ARAT) and Fugl-Meyer Assessment for Upper-Limb (FMA), and the neurological changes were assessed using resting-state functional magnetic resonance imaging. Repeated-measure ANOVAs indicated that long-term motor improvement was found by both FMA (F_[2,26]_ = 6.367, *p* = 0.006) and ARAT (F_[2,26]_ = 7.230, *p* = 0.003). Seed-based functional connectivity analysis exhibited that significantly modulated FC was observed between ipsilesional motor regions (primary motor cortex and supplementary motor area) and contralesional areas (supplementary motor area, premotor cortex, and superior parietal lobule), and the effects were sustained after 6 months. The fALFF analysis showed that local neuronal activities significantly increased in central, frontal and parietal regions, and the effects were also sustained after 6 months. Consistent results in FC and fALFF analyses demonstrated the increase of neural activities in sensorimotor and fronto-parietal regions, which were highly involved in the BCI-guided training.

**Clinical Trial Registration:** This study has been registered at ClinicalTrials.gov with clinical trial registration number NCT02323061.

## Introduction

Stroke survivors require high demand in rehabilitation and long-term care services, especially for upper extremity motor function (Norouzi-Gheidari et al., 2012). Fortunately, the existence of neuroplasticity, which characterizes the potential of modifying the size of cortical receptive field or motor output modules in response to altered synaptic input (Seitz et al., 1995), makes the development of various stroke rehabilitation methods possible.

Brain-computer interface (BCI)-guided training therapy has been promoted as a post-stroke motor rehabilitation training tool. It is designed to enhance motor recovery by modulating sensorimotor activity through repetitive practice with corresponding feedback or reward, thereby modifying the neuronal activity (Biasiucci et al., 2018; Remsik et al., 2019). In practice, BCI has been developed to translate brain activities into control signals of corresponding external execution devices such as robots, orthosis, and functional electrical stimulation (FES) (Soekadar et al., 2015; Cho et al., 2019; Mrachacz-Kersting et al., 2019; Mane et al., 2020). Therefore, combining the BCI system with a unilateral robotic hand technology makes it possible for stroke subjects to control the robotic hand with his/her brain signals, in order to restore the paretic hand function by promoting neuroplasticity and facilitating motor relearning (Frolov et al., 2017; Carino-Escobar et al., 2019). Clinical evidence showed that BCI-guided training elicits clinically significant and long-lasting motor recovery in chronic stroke survivors (Biasiucci et al., 2018; Ramos-Murguialday et al., 2019). A meta-analysis also suggested that BCI technology could be a more effective intervention for post-stroke upper-limb rehabilitation than other conventional therapies (Cervera et al., 2018). Despite the promising findings achieved, the underlying neurophysiological mechanisms induced by BCI-guided training for chronic stroke have not been thoroughly investigated. Besides, most existing BCI-guided robot-assist training studies adopted proximal joint upper limb training strategy (e.g., reaching and retrieving), such as in (Várkuti et al., 2012; Ramos-Murguialday et al., 2013), while our study applied robot hand on the distal joint of the upper-limb. Recently, studies comparing distal and proximal robot-assisted training therapies showed that distal training exhibited better performance than proximal training in the whole upper-limb (Hsieh et al., 2018; Qian et al., 2019).

The functional magnetic resonance imaging (fMRI) could be an essential tool to understand the effects of rehabilitation therapies on neuroplasticity. It is one of the most commonly used neuroimaging tools for assessing the cortical modulations in stroke (Kimberley et al., 2008). Resting-state fMRI (rs-fMRI) measures the blood oxygen level-dependent (BOLD) signal at the resting-state, which maps the functional organization of the brain (Van Essen et al., 2012). Functional connectivity (FC) calculates the temporal dependency of neuronal activation patterns in anatomically separated brain regions, and it is the most commonly used index in rs-fMRI studies (Van Den Heuvel and Hulshoff Pol, 2010). FC gives valuable information in the network-wide effects of stroke by providing great insight into network dysfunction and functional reorganization (Carter et al., 2012). It is suitable to investigate multiple distributed networks that were damaged by stroke and how connectivity patterns may be reorganized after recovery (Grefkes and Fink, 2014). In addition to provide a way to quantify neural activities across the whole brain, the fractional amplitude of low-frequency fluctuations (fALFF) reflects a different aspect of the BOLD signal, measuring the power of low-frequency fluctuations (Zuo et al., 2010). Lower frequency fluctuations allow us to study the amplitude of regional neuronal activity, which is an indication of local metabolic changes associated with the BOLD signal across the whole brain (Chen et al., 2015). fALFF analysis has been used to study post-stroke depression (Egorova et al., 2017) and motor recovery (Wang et al., 2020). However, few studies have combined FC and fALFF in investigating neuroplasticity changes induced by motor rehabilitation after stroke. These two measurements might provide complementary information as well as further validation for each other, which would make the evaluation more comprehensive. A comprehensive exploration in the whole-brain level is needed to fill the gap.

The hemispheric changes of resting-state functional connectivity and activation pattern shift during motor task after BCI-guided robot hand training have been demonstrated by our previous studies (Khan et al., 2020; Yuan et al., 2020). In this study, we aim at exploring the whole-brain neuroplasticity changes using rs-fMRI. Motor imagery studies have consistently disclosed activity in cortical and subcortical motor areas, which substantially overlap the neural substrates of motor execution (Hanakawa et al., 2008). Besides, motor imagery also involves some distinctive regions in the frontal and parietal regions which are not involved in motor execution (Hanakawa et al., 2008; Sharma et al., 2009). Therefore, we hypothesize that BCI-guided training could boost beneficial functional activity dependent plasticity to attain clinically important outcomes, through the contingency between suitable motor-related cortical activity and the afferent feedback. We believe that there should be functional reorganization within the sensorimotor and frontoparietal regions involved in the BCI-guided upper-limb training, which might account for the clinical improvement in the upper-limb function. We also expect the FC and the fALFF to show complementary results and to validate each other, since they represent different aspects of the fMRI data. Furthermore, we also tried to explore the neuroplasticity changes in a 6-month follow-up session to investigate whether the neuroplasticity changes could be maintained.

## Materials and Methods

### Subjects

Fourteen chronic stroke survivors (13 males, mean age = 54 ± 8 years) with right (*n* = 9) or left (*n* = 5) hemisphere impairment were recruited from the local community. The inclusion criteria were (1) first-ever stroke, (2) onset of stroke diagnose more than 6 months, (3) a single unilateral brain lesion, (4) sufficient cognition and comprehensive ability to understand and perform corresponding tasks assessed by Montreal Cognitive Assessment (MoCA) with a score of >21, (5) moderate to severe motor dysfunctions for the paretic upper extremity (Fugl-Meyer Assessment for upper-extremity score <47) (Woodbury et al., 2013) and (6) no additional rehabilitation therapies applied to the subject during the intervention. Subjects with (1) aphasia, neglect, and apraxia, history of alcohol, drug abuse, or epilepsy, (2) severe hand spasticity, (3) hand deformity and wound, (4) bilateral infracts, uncontrolled medical problems, and (5) serious cognitive deficits were excluded. The study was approved by the Joint Chinese University of Hong Kong-New Territories East Cluster Clinical Research Ethics Committee and all subjects signed written consent before any experiments started. This study has been registered at https://clinicaltrials.gov with clinical trial registration number NCT02323061.

Fugl-Meyer Assessment for upper-extremity (FMA) (Fugl-Meyer et al., 1975) and Action Research Arm Test (ARAT) (Lyle, 1981) were used to assess the motor function of the paretic upper limbs for all stroke subjects before (Pre), immediately after (Post) and 6 months after the intervention (Post6month) respectively. ARAT measures the affected upper limb's ability to reach, grasp, manipulate, and release objects which are regularly encountered during activities of daily living, with a maximum score of 57. FMA measures the motor function of the whole upper limb, with a maximum score of 66. Both assessments are widely used in upper-extremity rehabilitation.

### Training System and Intervention Protocols

During the training, subjects were asked to imagine the action of either grasping or releasing a cup following the instruction (generated by Psychophysics Toolbox 3.0, http://psychtoolbox.org/) displayed on the monitor. The task of motor imagery of opening and grasping the paretic hand is frequently used in our daily life, however, it is very challenging for stroke survivors. Through this task, the alpha suppression is detectable in the motor regions, according to previous studies (Neuper et al., 2009). This task is also widely adopted in other motor imagery studies (Neuper et al., 2009; Pichiorri et al., 2015). Each subject's electroencephalography (EEG) signals were acquired using a portable signal acquisition system (g.LADYbird, g.Tec Medical Engineering, GmbH, Austria) with 16 electrodes covering the motor-related regions in both ipsilesional and contralesional hemispheres (located at C1, C2, C3, C4, C5, C6, Cz, FC1, FC2, FC3, FC4, FCz, CP1, CP2, CP3, and CP4 according to the international 10–20 system). Impedances of electrodes were kept below 5 kΩ. EEG signals were referenced to the unilateral earlobe, the ground at Fpz, and sampled at 256 Hz. To remove artifacts and power line noise, a 2–60 Hz bandpass filter and 48–52 Hz notch filer were utilized in the real-time. All the channels were used to generate the dynamic potential topography of the whole brain for the trainer to inspect the state of each subject. An exoskeleton robot hand (Tong et al., 2013) was used to assist the paretic hand to grasp and open. From the fully extended position to the fully flexed position, the fingers assembly provided 55 degrees and 65 degrees range of motion (ROM) for the metacarpophalangeal (MCP) and proximal interphalangeal (PIP) finger joints, respectively. The robot hand assisted the subjects to open and grasp their paretic hand, which was very challenging by themselves. The biofeedback was easily sensible and functionally meaningful, resulting in providing rich sensory inputs via the natural afferent pathways in the real-time. We kept the robot hand's power assistance consistent throughout the 20-session training.

**Figure 1:**
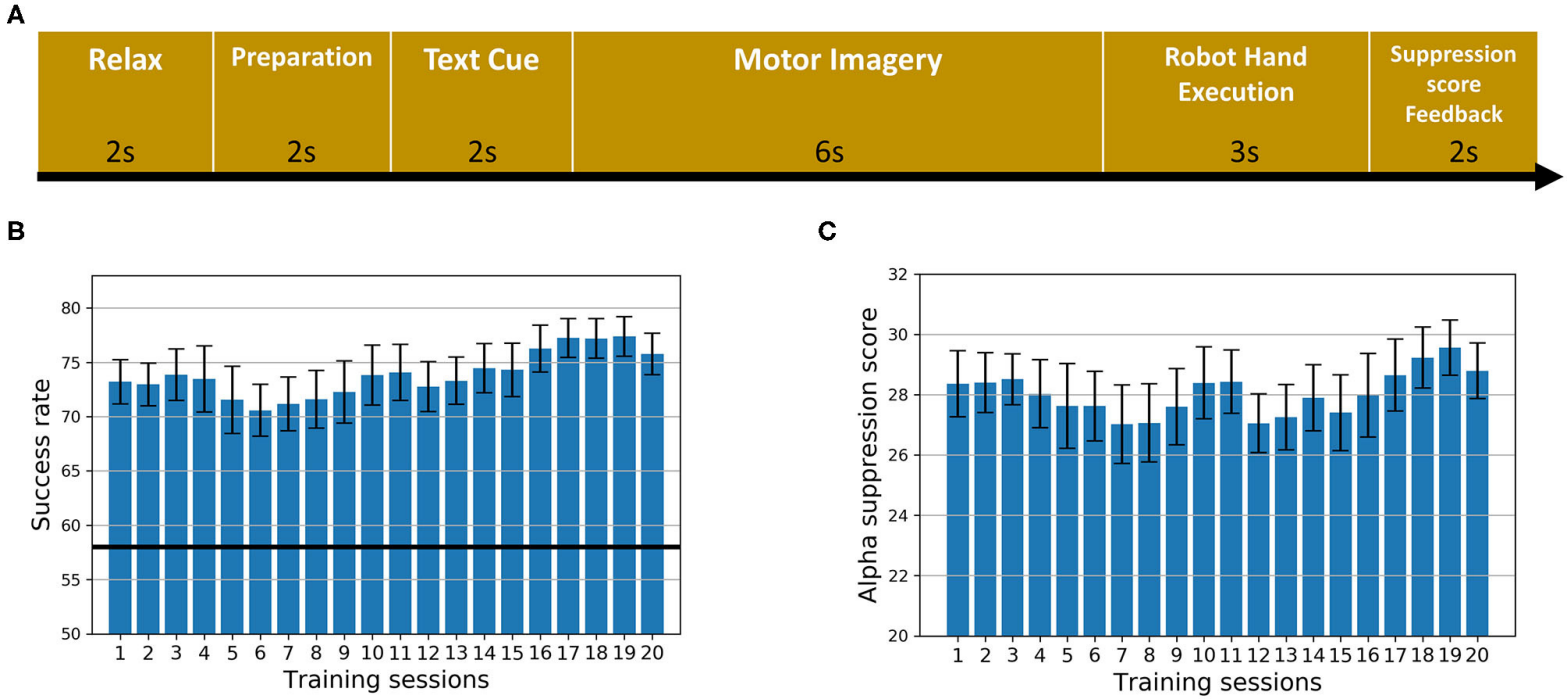
The intervention. **(A)** The sequence of the training paradigm. **(B)** The average success rate of all training trials across 20 sessions for all subjects. Error bars stand for standard errors. The dark black line stands for the chance level. **(C)** The average α suppression score of all training trials across 20 sessions for all subjects. Error bars stand for standard errors.

The sequence of the training paradigm is illustrated in Figure 1A. During each trial, the subject was asked to relax for 2 s followed by a white cross for 2 s to remind them to get ready. A text cue of “hand grasp” or “hand open” was then displayed for 2 s to instruct the subject to imagine the corresponding action as if performed by his/her affected hand. After that, a video clip with a duration of 6 s showing either the action of grasping or releasing a cup was displayed simultaneously for guidance. The trigger to the robot hand was then sent based on the α suppression (8–13 Hz) of the EEG signal during the motor imagery. In the following 3 s, the robot hand assisted the subject in completing the grasping/opening task. Afterwards, the α suppression score as a feedback was displayed on the screen for 2 s to motivate the subject to achieve a higher score in the subsequent trials. Finally, a 2 s rest was given to the subject before the next trial started. The text cue of “hand grasp” and “hand open” appeared alternately. To compute the α suppression score, the EEG signal from either the C3 or C4 channel was chosen, according to the lesion side, and transformed to the frequency domain through fast Fourier transform with a Hanning window. The mean power was calculated in the α band (8–13 Hz) and compared with the baseline before motor imagery. Specifically, the α suppression score (α_*S*_) was calculated as follows (Ono et al., 2014):

$$\begin{array}{l} \alpha_{S}=-\frac{P_{MI}-P_{rest}}{P_{rest}}\times 100\% \end{array}$$

where *P*_*MI*_ represented the mean power of α band during motor imagery and *P*_*rest*_ stood for the mean α power during resting state. In our study, we predefined that the robot hand would apply a mechanical force to assist the subject in completing the hand opening or grasping tasks if the suppression score α_*S*_ exceeded 20% based on the previous study (Perry and Bentin, 2009). The success rate was defined as the percentage of correctly detected trials at each session. All subjects received a 20-session BCI robot hand training with an intensity of 3–5 sessions per week and completed the whole process within 5–7 weeks. During each training session, the subject was required to perform 100 repetitive hand opening/grasping tasks and the intermittent breaks were given at every 10 repetitions to avoid fatigue.

### Data Acquisition

MRI scans were acquired for all the 14 subjects at Pre and Post sessions. Ten subjects had a Post6month session scan, while four subjects did not attend the Post6month session. A 3T Philips MR scanner (Achieva TX, Philips Medical System, Best, Netherlands) with an 8-channel head coil was used to acquire high resolution T1-weighted anatomical images (TR/TE = 7.47/3.45 ms, flip angle = 8, 308 slices, voxel size = 0.6 × 1.042 × 1.042 *mm*^3^) using a T1-TFE sequence (ultrafast spoiled gradient echo pulse sequence), and BOLD fMRI images (TR/TE = 2,000/30 ms, flip angle = 70°, 37 slices/volume, voxel size = 2.8 × 2.8 × 3.5 *mm*^3^) using a FEEPI sequence (gradient-echo echo-planar-imaging sequence). The sequences were displayed using EPrime 2.0 (Psychology Software Tools, PA USA). During the acquisition of rs-fMRI data, subjects were presented with a white cross in a black background and instructed to rest while focusing on the fixation cross. One rs-fMRI block lasted for 8 min.

### Assessment Score and Training Performance Analysis

Repeated measure analysis of variance (ANOVA) at time level (Pre, Post, and Post6month) were applied to examine whether the FMA and ARAT scores improved after the intervention. Paired *t*-tests were used as *post-hoc* tests to examine significant changes in different combinations of three time-points for the FMA and ARAT scores. Normality of the data was checked using Kolmogorov-Smirnov tests and the results showed the data were normally distributed. Bonferroni corrections were used to adjust for multiple comparisons. Statistical analyses were performed using SPSS 25.0 (IBM SPSS Statistics, NY, US) with the significance level set at corrected *p* < 0.05. Moreover, the minimal clinically important difference (MCID) was also calculated to reflect the clinical significance by setting minimal changes in clinical assessments. The MCID of FMA is 4.25 (Page et al., 2012) and the MCID of ARAT is 5.7 in chronic stroke (Van Der Lee et al., 2001). Mean success rate and suppression score of training trials across 20 sessions for all the subjects were calculated, respectively.

### fMRI Analysis Preprocessing

The rs-fMRI data were preprocessed using DPARSF toolbox. The first 10 volumes were discarded to assure the remaining volumes of fMRI data were at magnetization steady state. The remaining volumes were corrected with slice timing and realigned for head motion correction. Nuisance variables were then regressed out, including white matter, cerebrospinal fluid (CSF), global mean signal, and Friston 24 head motion parameters (Friston et al., 1996). To further control for head motion, the scrubbing process was performed for the volumes with framewise displacement (FD) value exceeding 0.3 (Power et al., 2012). If over 25% of all the volumes exceed the FD threshold, the data for this subject would be discarded, and no subject was discarded in the rs-fMRI analysis. Then the functional dataset was aligned to the anatomical dataset. Detrending and temporal band-pass filtering (0.01–0.1 Hz) (Zuo et al., 2010) were performed to remove higher frequency physiological noise and lower frequency scanner drift. Subsequently, the functional images were spatially normalized to the Montreal Neurological Institute (MNI) template, resliced to 2 × 2 × 2 *mm*^3^ voxels, and smoothed with a Gaussian kernel with a full-width at half-maximum (FWHM) of 6 mm. To perform group statistical analysis later, subjects with left-hemispheric lesions were flipped along the midsagittal plane using MRIcron (www.mccauslandcenter.sc.edu/mricro/mricron), so that the lesions of all subjects were in the right hemisphere. For the preprocessing steps for the fALFF analysis, the bandpass filter was not applied.

### Seed-Based FC Analysis

We did a seed-based whole-brain analysis with the seed at the ipsilesional primary motor cortex (iM1) and supplementary motor area (iSMA), and the seed locations were (38, −22, 56) and (8, −8, 57) in MNI space, respectively. The seeds were defined as spherical balls with a radius of 5 mm in MNI standard space. The average time course of the BOLD signals within the seeds was used to calculate the FC with every other voxel in the brain, producing maps of FC with the seeds. A paired *t*-test was carried out between each pair of sessions for all the seed-based FC maps. Multiple comparisons were corrected using Gaussian random field theory at the cluster level (minimum z > 2.7; cluster-wise significance: *p* < 0.05, corrected) (Chen et al., 2018). All the analyses for seed-based FC and paired *t*-test were carried out in DPARSF toolbox (Yan et al., 2016).

### fALFF Analysis

The fALFF values were computed on preprocessed data using the DPARSF software (Yan et al., 2016). DPARSF has in-built fast Fourier transform functions to convert time series data to the frequency domain and calculate the power spectrum. Briefly, on a voxel-by-voxel basis, the time course was converted into the frequency domain using a Fast Fourier Transform, the square root of the power spectrum was computed, and the average of the amplitudes in the range of 0.01–0.1 Hz was then calculated to obtain the ALFF (Zou et al., 2008; Zuo et al., 2010). Dividing each voxel's ALFF value by the amplitudes of the entire detectable frequency range (0–0.55 Hz) yields the fALFF (Zou et al., 2008). All analyses were performed at the whole-brain level. A paired *t*-test was carried out between each pair of sessions for results from fALFF analysis.

## Results

### Assessment Scores and Training Performance

Subject demographic and assessment scores are shown in Table 1. The repeated measure ANOVA on FMA scores with time (Pre, Post, and Post6month) as within-subject factor indicated that a significant effect of time (F_[2,26]_ = 6.367, *p* = 0.006) was observed. *Post-hoc* tests indicated that there were significant increases in FMA scores between Pre and Post (*p* = 0.017, Bonferroni corrected) as well as between Pre and Post6month (*p* = 0.034, Bonferroni corrected). No significant change was found between Post and Post6month (*p* = 1.00, Bonferroni corrected). The repeated measure ANOVA on ARAT scores with time (Pre, Post, and Post6month) as within-subject factor indicated that a significant effect of time (F_[2,26]_ = 7.230, *p* = 0.003) was observed. *Post-hoc* tests indicated that there were significant increases in ARAT scores between Pre and Post (*p* = 0.015, Bonferroni corrected), marginally significant between Pre and Post6month (*p* = 0.055, Bonferroni corrected). No significant change was found between Post and Post6month (*p* = 0.879, Bonferroni corrected). The result indicated that the BCI robot hand training was able to promote motor recovery with a long-term effect. For FMA scores, 43% of the subjects achieved the MCID at Post and 36% of the subjects achieved the MCID at Post6month. For ARAT scores, 36% of the subjects achieved the MCID at Post and 29% of the subjects achieved the MCID at Post6month. For suppression score at the ipsilesional motor area (Figure 1C), a slightly increasing trend from the beginning to the end of all the sessions could be observed, with the average of 28.19% for the first five sessions to 28.85% for the last five sessions. For the success rate of training trials (Figure 1B), an increasing trend was observed, with the average of 73.01% for the first five sessions to 76.78% for the last five sessions. The chance level was 58% (Müller-Putz et al., 2008). The results on the performances of motor imagery tasks showed that the subjects were improving with the increased number of training sessions.

**Table 1:** Demographic and assessment scores. Fourteen chronic stroke subjects participated in this study.

						**ARAT (max. score: 57)**			**FMA (max. score: 66)**		
**Subjects**	**Age range**	**Gender**	**Lesion Locations**	**Stroke Onset Time (years)**	**Stroke Type**	**Pre**	**Post**	**Post 6month**	**Pre**	**Post**	**Post 6month**
1^#^	45–49	M	R MFG, SFG, precentral supramarginal, SMA	1	I	3	21	19	19	34	28
2	65–69	M	L insula, putamen, IFG, temporal pole	8	H	10	21	15	22	27	32
3	65–69	M	R insula, ITG, IOG, putamen	1	H	8	15	26	13	16	27
4	60–64	M	R insula, putamen, IFG, rolandic operculum	3	I	4	6	8	16	14	18
5	45–49	M	R ITG, MTG, STG, MOG, angular, supramarginal	0.7	H	16	17	17	17	25	25
6	60–64	M	L PLIC, putamen, insula,postcentral,SFG	11	I	15	14	11	22	24	24
7	55–59	M	R insula, IFG, rolandic operculum	6	I	12	21	20	13	23	20
8	40–44	M	R insula, rolandic operculum, IFG, STG, putamen, temporal pole	5	H	9	14	10	15	17	16
9^#^	50–54	F	L insula, rolandic operculum, putamen	3	H	19	23	22	34	34	37
10	40–44	M	R insula, MTG, STG, temporal pole, putamen, rolandic operculum	3	H	11	14	13	17	20	20
11^#^	55–59	M	L insula, IFG, putamen	5	H	10	12	8	28	33	24
12^#^	50–54	M	L putamen, caudate nucleus	1	I	15	13	16	24	22	22
13	55–59	M	R putamen,temporal pole, IFG, insula, rolandic operculum	7	I	14	19	17	20	25	21
14	45–49	M	R insula, putamen	1	H	12	33	18	34	37	35
					Mean ± Std	11.3 ± 4.3	**17.3** **±** 6.2^*^	15.7 ± 5.1	21.0 ± 6.7	**25.1** **±** 7.0^*^	**25.0** **±** 6.1^*^

# Subjects that did not have Post6month MRI scan.

* Significant increase compared with the Pre (p < 0.05).

*ARAT, Action Research Arm Test; F, female; FMA, Fugel-Meyer Assessment for upper limb; H, hemorrhage stroke; I, ischemic stroke; IFG, Inferior frontal gyrus; IOG, Inferior occipital gyrus; ITG, Inferior temporal gyrus; L, left; M, male; MFG, Middle frontal gyrus; MOG, Middle occipital gyrus; MTG, Middle temporal gyrus; PLIC, Posterior limb of the internal capsule; R, right; SFG, superior frontal gyrus; SMA, Supplementary motor area; STG, Superior temporal gyrus*.

### Seed-Based FC Analysis

**Figure 2:**
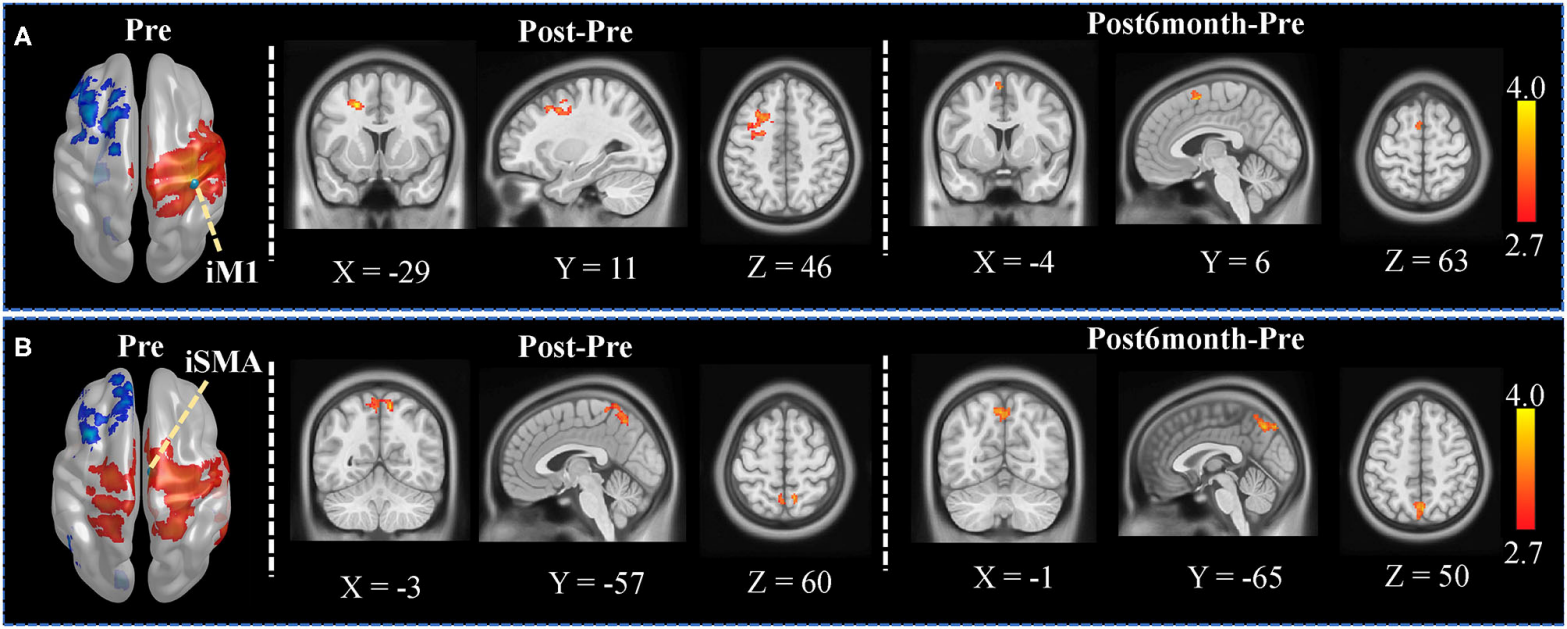
Seed-based whole-brain analysis results. **(A)** The left panel showed the FC map in the Pre session when the seed was set at iM1. Voxels with z > 2.7 were shown. The iM1 seed was denoted as a green sphere in the figure. The color-coded area illustrates the significant clusters found in Post (contralesional premotor area) and Post6month (contralesional SMA). The white numbers beside the images represent the coordinate in MNI space. **(B)** The left panel showed the FC map in the Pre session when the seed was set at iSMA. Voxels with z > 2.7 were shown. The iSMA seed was denoted as a green sphere in the figure. The color-coded area illustrates the significant clusters found in Post (bilateral SPL) and Post6month (bilateral SPL). The white numbers beside the images represent the coordinate in MNI space.

Seed-based whole-brain FC was explored between each pair of sessions with the seed set at iM1 and iSMA. The FC map in the Pre session with the iM1 seed was shown in the left panel of Figure 2A. Significant increased FC was found between iM1, and the contralesional premotor cortex as well as part of SMA (Figure 2A), when comparing Post and Pre sessions; significant increased FC was found between iM1 and contralesional SMA (Figure 2A) when comparing Post6month and Pre sessions. The FC map in the Pre session with the iSMA seed was shown in the left panel of Figure 2B. Significant increased FC was found between iSMA with bilateral superior parietal lobe (SPL) when comparing Post and Post6month to Pre session (Figure 2B).

### fALFF Analysis

**Figure 3:**
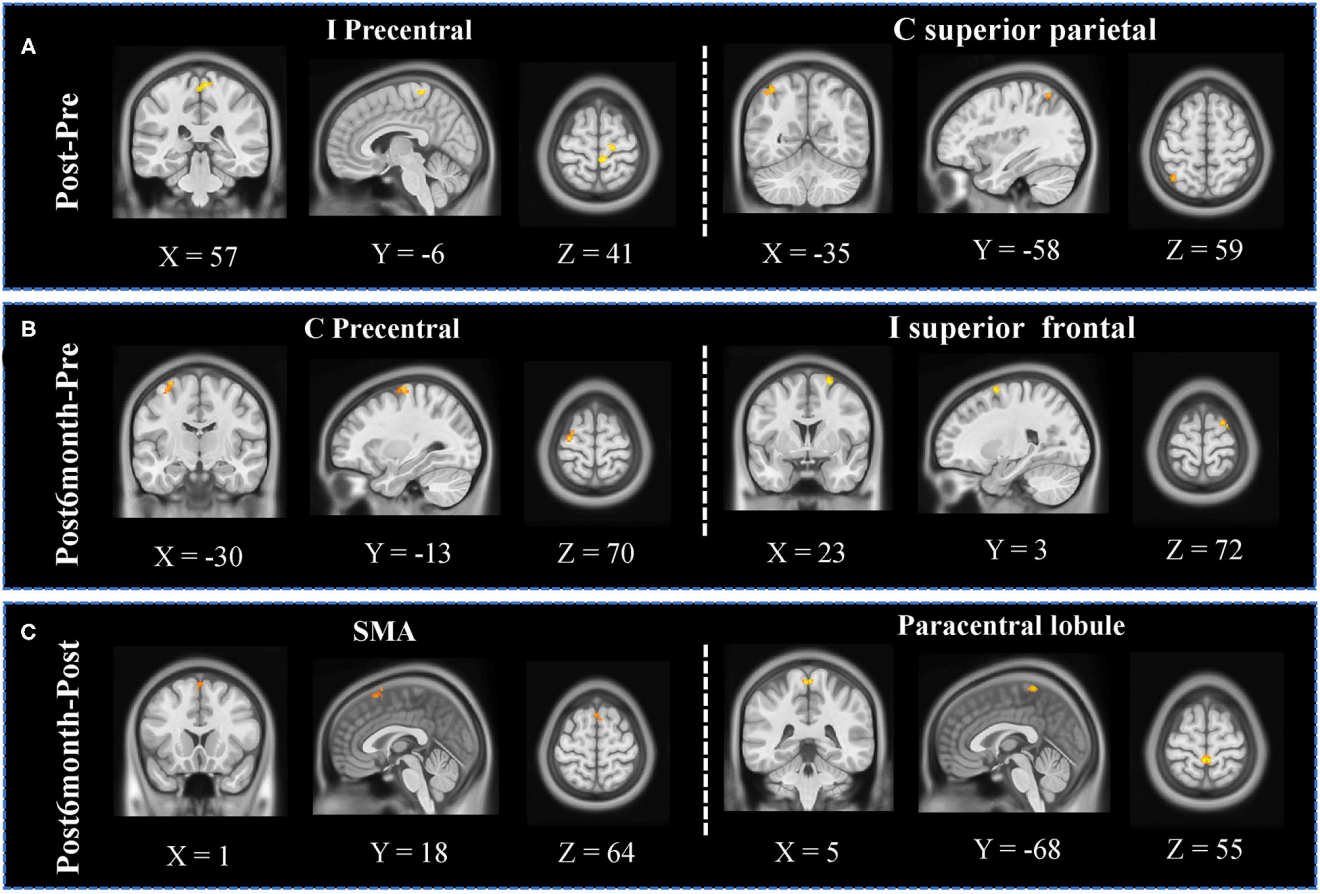
fALFF analyses results. **(A)** Significant increased clusters were observed in the ipsilesional precentral area and contralesional superior parietal lobule when comparing Post and Pre sessions. **(B)** Significantly increased clusters were observed in the contralesional precentral area and ipsilesional superior frontal area when comparing Post6month and Pre sessions. **(C)** Significantly increased clusters were observed in bilateral SMA and paracentral lobule when comparing Post6month and Post sessions.

Significantly increased fALFF was observed in the ipsilesional precentral area and superior parietal lobule (Figure 3A) when comparing Post and Pre sessions; significantly increased fALFF was observed in the contralesional precentral area and ipsilesional superior frontal area (Figure 3B) when comparing Post6month and Pre sessions; significantly increased fALFF was observed in bilateral SMA and paracentral lobule (Figure 3C) when comparing Post6month and Post sessions.

## Discussion

This study explored the effects of BCI-guided robot hand training on the paretic hand in chronic stroke survivors by providing repetitive exercise with integrated sensorimotor feedback. The subjects showed upper-limb motor function improvement, as reflected by the FMA and the ARAT scores, after the 20-session training and these improvements were sustained 6 months after the intervention. Moreover, this study also revealed the neuroplasticity changes after the intervention. The FC between iM1 and contralesional premotor cortex and SMA significantly increased immediately after the 20-session training; The FC between iSMA and bilateral SPL also significantly increased immediately after the 20-session training. Besides, the fALFF analysis showed that local neuronal activities significantly increased in central, frontal, and parietal regions. Our study demonstrated the modulated neuroplasticity changes introduced by the BCI-guided robot hand training immediately and 6 months after the intervention.

Stroke survivors experience spontaneous recovery within the first few months after stroke onset and they then become clinically stable with hand weakness (Tombari et al., 2004; Kwakkel et al., 2006). BCI-guided robot therapy has shown the potential to restore motor function and to improve rehabilitation outcomes after stroke or spinal cord injury (Frolov et al., 2017). The feedback facilitates the appraisal of performance by enforcing the sensory aspect in the sensorimotor loop and thereby restoring the action-perception coupling (Daly and Wolpaw, 2008; Van Dokkum et al., 2015). The feedback in this study is the hand opening and grasping movement assisted by the robot hand, which is easily sensible and functionally meaningful. It provides rich sensory inputs through the natural afferent pathways in real-time. Studies have reported that BCI-guided training helps promote upper-limb motor function more than conventional therapies, as well as induces functional reorganization in the brain (Ramos-Murguialday et al., 2013; Biasiucci et al., 2018). According to these studies, no significant improvement was found in upper-limb FMA scores in the groups who received random functional electrical stimulation (FES) (Biasiucci et al., 2018) or received random robotic orthosis feedback (Ramos-Murguialday et al., 2013). Our results further validate the potential of BCI-guided intervention in promoting hand function recovery for persons with chronic stroke.

In our study, the seed-based whole-brain analysis showed that the FC between iM1 and contralesional premotor cortex and SMA was significantly increased after the 20-session training. Studies have suggested that premotor cortex and M1 play a crucial role during motor-imagery as well as during motor execution tasks (Bajaj et al., 2015). The crucial role of the SMA in motor recovery has already been demonstrated in previous fMRI studies (Loubinoux et al., 2003; Tombari et al., 2004). A longitudinal fMRI study indicated that the connectivity of the iM1 with the contralesional regions including SMA at the early stage of stroke was positively correlated with motor improvement (Park et al., 2011). A concurrent TMS-fMRI study indicated that the contralesional premotor area might support the residual motor function following stroke and have an increasing influence on the survived sensorimotor cortex in the ipsilesional hemisphere on subjects with more impairment (Bestmann et al., 2010), which may be the potential reason for the increased FC between the iM1 and premotor area after training. Besides, the FC between iSMA and bilateral SPL also increased, according to the current study. During both motor imagery and motor execution, SPL is activated (Guillot et al., 2012), although not exactly overlapping with each other. The MI-based BCI training in our study decoded the sensorimotor EEG signals to trigger the robotic hand, offering rich afferent neural feedback. The whole training process involved these modulated brain regions and hence, promoting motor relearning during the training.

Investigating different dimensions of resting-state BOLD activity is important, as differences may lie not only in the patterns of connectivity but also the power of local neuronal activity. Apart from the FC, we also investigate the changes in fALFF. While FC measures the temporal correlation between the activations at two given regions, the fALFF measures the power of low-frequency fluctuations, which allows us to study the amplitude of regional neuronal activity. As different frequency bands originate from different neural sources, they could relate to different aspects of brain processing. With the oscillation in the range that is most closely related to gray matter signal, it shows the most extensive change after stroke (Zhu et al., 2015; Wang et al., 2020). A longitudinal study on stroke subjects showed that stroke survivors exhibited lower amplitude of oscillations in comparison to healthy controls in the subacute stage, and those same subjects showed a recovery of the oscillations, reaching near equivalence to the healthy controls (La et al., 2016). Another recent study on chronic stroke subjects suggested that motor imagery training plus conventional rehabilitation therapy-induced increased fALFF in the ipsilesional inferior parietal lobule, which is positively correlated with upper-limb motor function improvement (Wang et al., 2020). We found that the fALFF significantly increased in the ipsilesional precentral area and superior parietal lobule (SPL) immediately after the intervention, moreover, increases in the contralesional precentral area and ipsilesional superior frontal area were observed 6 months after the intervention. Interestingly, the significantly modulated regions in the fALFF analysis were quite consistent with the results from the FC analysis, which further validated each other. Other studies have also reported that brain regions in the fronto-parietal network were highly related to motor imagery BCI training (Cincotti et al., 2012), and correlated with the performance of MI-BCI (Zhang et al., 2016). Pichiorri et al. indicated that the BCI-supported MI training group showed more significantly increased connections over the MI-only group between ipsilesional motor area and contralesional frontal and parietal areas in the beta band of resting-state EEG data, which were speculated as related to training effects (Pichiorri et al., 2015). In our study, significant modulated neural activities were observed not only in central regions but also in frontal and parietal regions, which were highly specific to BCI-guided robot hand training. These findings might suggest that the intervention could modulate the brain activities not limited to the sensorimotor network, but also in other regions associated with motor imagery and robot hand training.

This pilot study has shown the potential of the intervention for promoting hand function recovery and its long-term effect in chronic stroke survivors. fMRI might be able to provide insights into neural mechanisms underlying the recovery of motor function and reorganization of brain networks. Our findings provide some insights into the effects on neuroplasticity changes induced by the BCI-guided upper-limb training. Several limitations need to be stated in this study. First of all, the current study lacks a control condition. In order to differentiate the effects brought by volitional BCI based training and pure robot hand training, a control condition is needed. Second, the sample size is relatively small which might limit the generalization power. More subjects should be recruited to validate and extend the findings of this study.

## Data Availability Statement

The raw data supporting the conclusions of this article will be made available by the authors, without undue reservation.

## Ethics Statement

The studies involving human participants were reviewed and approved by The Joint Chinese University of Hong Kong-New Territories East Cluster Clinical Research Ethics Committee. The patients/participants provided their written informed consent to participate in this study.

## Author Contributions

CL, KY, and RT made substantial contributions to data analysis and drafting the manuscript. CL and W-wW contributed to experimental design and data collection. PW, WC, and TL offered their expert advice in screening the subjects and interpreting the results. All authors contributed to the article and approved the submitted version.

## Conflict of Interest

RT is one of the inventors of the Hong Kong Polytechnic University-held patent for the hand exoskeleton robot which was used in this study. All authors, however, are of no financial relationship whatsoever for the submitted work with Rehab-Robotics Company Ltd., the company which manufactures the commercial version of the original device under a license agreement with the University.
